# Sustained inflammation during human T-lymphotropic virus type 1 infection: a wildfire contributing to disease progression

**DOI:** 10.3389/fmed.2025.1653384

**Published:** 2025-09-12

**Authors:** Saina Shegefti, Mahsa Alaei, Nazanin Ghahari, Roman Telittchenko, Shahin Bolori Hanafi, Stephane Isnard, Jean-Pierre Routy, David Olagnier, Julien van Grevenynghe

**Affiliations:** ^1^Institut national de la recherche scientifique (INRS), Centre Armand-Frappier Santé Biotechnologie, Laval, QC, Canada; ^2^Meakins-Christie Laboratories, Research Institute of the McGill University Health Centre, Montreal, QC, Canada; ^3^McGill University Health Centre, Montreal, QC, Canada; ^4^Department of Biomedicine. Aarhus University, Aarhus C, Denmark

**Keywords:** HTLV-1, HTLV-1 tax protein, inflammation, NF-κB signaling pathway, cytokine/chemokine, ATLL, HAM-TSP, asymptomatic carriers

## Abstract

Human T-lymphotropic virus type 1 (HTLV-1) is a retrovirus affecting 10–20 million people worldwide. While many carriers remain asymptomatic, HTLV-1 infection can trigger intense inflammatory responses which are defined by the sustained release of pro-inflammatory cytokines and chemokines. Central to this process is the HTLV-1 encoded Tax oncoprotein, a viral regulator that drives uncontrolled inflammation by hijacking multiple cellular signaling pathways, such as the RelA/NF-κB signal transduction pathway. CD4 T-cells are the primary targets of Tax-mediated transformation, undergoing uncontrolled proliferation and significantly contributing to chronic immune activation seen in HTLV-1-associated diseases. However, highly activated CD4 T-cells are not alone in fueling this inflammatory “wildfire.” Other immune cells, including CD8 T-cells, monocytes, macrophages, dendritic cells, and neutrophils, also play critical roles in exacerbating the inflammatory milieu. These cells, in conjunction with CD4 T-cells, release a barrage of pro-inflammatory cytokines (IL-1α/β, IL-2, IL-6, IL-12, IL-17, TNF-α/β, and IFN-γ) and chemokines (MCP-1, MIP-1α/β, RANTES, MCP-3, IL-8, CXCL9, CXCL10, and CXCL11), all of which are perpetuating the cycle of immune activation and tissue damage. This hyper stimulated immune response contributes to HTLV-1 replication/dissemination and can lead to the development of adult T-cell leukemia/lymphoma (ATLL) and HTLV-1-associated myelopathy/tropical spastic paraparesis (HAM-TSP). Despite existing treatments aimed at controlling viral replication, the persistent inflammation in HTLV-1-infected individuals even in asymptomatic carriers (ACs) remains a major challenge, suggesting that targeting these pro-inflammatory responses may be another mandatory therapeutic strategy. In this context, this short-review focuses on the key immune responses that drive HTLV-1-associated inflammation and explores how these high pro-inflammatory responses contribute to the development of HTLV-1-related complications, including HAM-TSP, ATLL, and other associated inflammatory diseases during chronic viral infection.

## 1 Introduction: the need to develop new strategies for counteracting HTLV-1

The infection with the human T-cell leukemia virus type 1 (HTLV-1), the only known human oncogenic retrovirus, has been recently estimated to affect up to 20 million people worldwide. It is predominantly spreading across endemic regions in Japan, Africa, Asia, the Caribbean, Central/South America, the Middle East and includes the Australo-Melanesia area (1–3). The virus is transmitted through the bodily fluids of infected individuals, primarily breast milk, blood, and semen (4, 5). Although approximately 90% of the infected individuals remain asymptomatic carriers during their lives, chronic infection with HTLV-1 can result in multiple severe pathologies; these include the adult T-cell leukemia/lymphoma (ATLL), an aggressive neoplasm of CD25^+^ CD4 T-cells in about 5% of infected individuals after a prolonged latent period of 30–50 years (2, 6). HTLV-1 infection is also the causative agent of inflammatory disorders, most notably HTLV-1-associated myelopathy/tropical spastic paraparesis (HAM/TSP) asides other afflictions, such as uveitis, a chronic inflammatory interstitial lung disease called cryptogenic fibrosing alveolitis (CFA), rheumatic syndromes and a high predisposition to glaucoma, sarcopenia, atherosclerosis, helminthic and bacterial infections (7–10). Currently, there are no prospects of functional vaccines for HTLV-1, screening of blood banks and there are no universal diagnostic tools in prenatal care settings. Existing treatments for ATLL and HAM/TSP are largely ineffective, thus emphasizing the urgent need for new targeted therapies (1, 11–14). A deeper understanding of how HTLV-1 infection impacts immune responses and persist in the host is a critical step for the development of these novel antiviral strategies. In this context, this short-review aims to provide a brief overview of the uncontrolled inflammatory responses reported in HTLV-1 infections and how they actively contribute to viral dissemination and disease development.

## 2 HTLV-1 infection causes strong and sustained inflammatory responses

**FIGURE 1:**
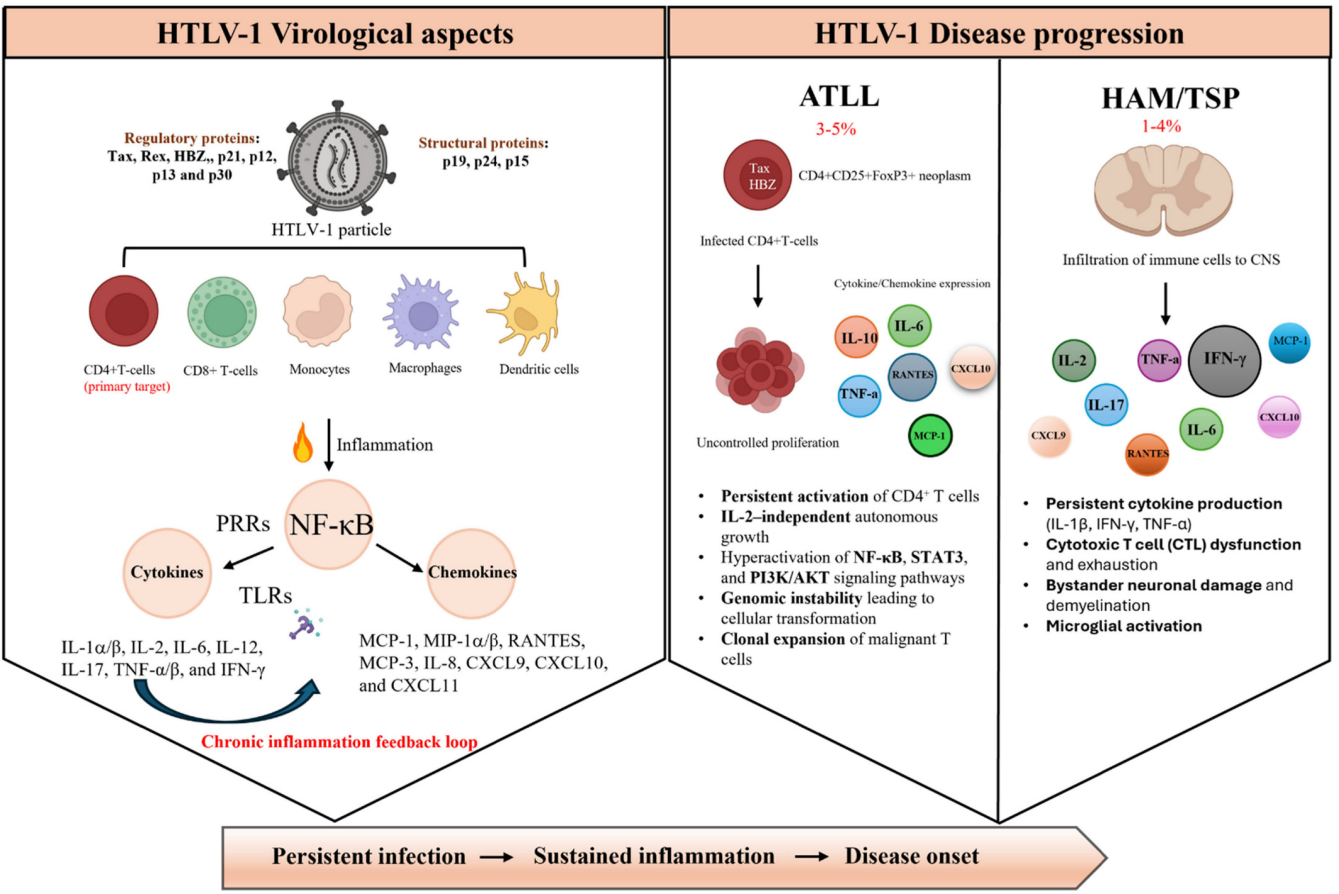
Schematic representation of HTLV-1 virological aspects and disease progression. (Left side) HTLV-1 infects multiple immune cell types, primarily CD4^1^ T cells, but also CDS^1^ T cells, monocytes, macrophages, and dendritic cells. Viral proteins (regulatory: Tax, Rex, HBZ, p21, pl2, p13, p30; structural: pl9, p24, p15) activate pattern recognition receptors (PRRs) and toll-like receptors (TLRs), leading to NF-xB-mediated production of pro-inflammatory cytokines and chemokines, establishing a chronic inflammation feedback loop. (Right side) Disease outcomes include adult T-cell leukemia/lymphoma (ATLL, 3%–5% of infected individuals) characterized by uncontrolled CD4^+^ T-cell proliferation, persistent activation of NF-KB, STAT3, and PT3K/AKT pathways, and clonal expansion; and HTLV-1- associated myelopathy/tropical spastic paraparesis (HAM/TSP, 1%–4% of infected individuals) involving central nervous system (CNS) infiltration, persistent cytokine production, CTL dysfunction, and neuronal damage. ATLL, adult T-cell leukemia/lymphoma; CNS, central nervous system; CTL, cytotoxic T lymphocyte; CXCL, C-X-C motif chemokine ligand; HBZ, HTLV-1 basic leucine zipper factor; HAM/TSP, HTLV-1-associated myelopathy/tropical spastic paraparesis; IITLV-l, human T-cell leukemia virus type 1; IFN, interferon; IL, interleukin; MCP, monocyte chemoattractant protein; M1P, macrophage inflammatory protein; NF-kB, nuclear factor kappa-light-chain-enhancer of activated B cells; PI3K/AKT, phosphoinositide 3-kinase/protein kinase B pathway; PRRs, pattern recognition receptors; RANTES, regulated upon activation, normal T cell expressed and secreted; STAT3, signal transducer and activator of transcription 3; TLRs, toll-like receptors; TNF-a, tumor necrosis factor alpha.

The immunopathogenesis of HTLV-1 is intriguing, since its lifelong persistence in the host determines a prolonged interaction between the virus and the immune system, which can ultimately contribute to the development of both ATLL and HAM/TSP conditions when inflammatory responses become uncontrolled. Although CD4 T-cells remain the main cell target for HTLV-1 (1, 15), the virus can also infect CD8 T-cells and immune cells of the myeloid lineage like dendritic cells (DCs), monocytes, and macrophages, altogether sustaining a strong poly-inflammatory milieu in the infected hosts due to HTLV-1 persistence (16–19). Although multiple causal factors during chronic HTLV-1 infection contribute to trigger the uncontrolled inflammatory responses, HTLV-1 Tax protein, the host innate sensing and high TNF-α release play a pivotal role in the process, mainly by constitutively inducing RelA/NF-κB signal transduction pathway in infected individuals (Figure 1 and Tables 1, 2).

**TABLE 1:** Pro-inflammatory cytokines and HTLV-1 infection, including associations with virus-related HAM-TSP and ATLL conditions.

Name	Aliases	Info in the context of HTLV-1 infection	Additional info	References
***pro-lnflammatory cytokines***				
IL-1 (α/β)	LAF	Tax-treated microglia cells secrete high protein levels for IL-lp	Cell supernatants (48 h of culture)	(39)
		Higher protein release of IL-ip in HAM-TSP vs. HCs	PBMCsupernatants (24 h of unstimulated culture)	(82)
		HTLV-1-infected CD4T-cells from HTLV-1^+^uveitis patients produced large amounts of IL-1	Infiltrating CD4T-cells in eyes	(91)
		High mRNA expression of both *ILIA* and *IL1B* in ATLLcells from Tax-transgenic mice	Expression in both Tax’ and Tax^+^cells	(105)
IL-2	Lymphokine2	Tax-treated MDDCs secrete IL-2cytokine in culture	Cell supernatants (24 h of culture)	(36)
		Higher mRNA levels for *IL2* in PBMCs from ATLL patients vs. HCs	Along with increased Nf-KB-related genes	(51)
		Neutralization of IL-2 decreases IFN-y levels in PBMC culture from ACs	PBMCsupernatants (24–48 h of culture)	(62)
		Higher plasma levels for IL-2 in HAM-TSP patients vs. ACs	Higher levels in HAM-TSP patients vs. ATLL	(67)
		High production and cell dependency to IL-2 of HTLV-l-infected CD4T-cells for proliferation	Contribution to cell transformation (after weeks of culture stimulation)	(98)
IL-6	Interferon beta-2	Tax-treated microglia cells secrete high protein levels for IL-6	Cell supernatants (48 h of culture)	(39)
		Higher plasma levels for IL-6 in ATLLpatients with aggressive cancer form vs. “indolent” form	Correlation between high plasma IL-6levels and shorter survival rates in ATLL	(67)
		Higher protein levels for IL-6 in both sera and CSF from HAM-TSP patients vs. ACs	Along with higher IL-6 activity	(69)
		Higher mRNA levels of *IL6* in neutrophils from HAM-TSP patients vs. ACs	Along with increased Nf-kB-related genes	(85)
		HTLV-l-infected CD4T-cells from HTLV-1^+^uveitis patients produced large amounts of IL-6	Infiltrating CD4T-cells in eyes	(91)
		Higher sera levels for IL-6 in ATLL patients vs. ACs and HCs	Correlation between high plasma IL-6 levels and ATLL severity	(100)
		High mRNA expression of both *IL6* in ATLL cells from Tax-transgenic mice	Expression in both Tax’ and Tax^+^ cells	(105)
IL-12		Tax-treated MDDCs secrete IL-12cytokine in culture	Cell supernatants (24/48 h of culture); induction in a Nf-KB-dependent manner	(36, 37)
		Higher proportion of IL-12-expressing monocytes and pDCs in HAM-TSP patients vs. ACs	PBMCs stimulated for 48 h of culture with TLR_7/8_ agonist (innate sensing)	(84)
		Higher mRNA levels of *IL17* in neutrophils from HAM-TSP patients vs. ACs	Along with increased Nf-kB-related genes	(85)
IL-17	IL-17A; CTLA-8	Higher mRNA levels of *IL17* in PBMCsfrom ATLLpatients vs. HCs	Along with increased Nf-KB-related genes	(51)
		Higher plasma levels for IL-17 in HAM-TSP patients vs. ACs	Higher levels in HAM-TSP patients vs. ATLL	(67)
		Higher proportion of IL-17-expressing CD4T-cells in HAM-TSP patients vs. ACs and HCs	PBMC culture with 3 days of cell stimulation	(79)
		Increased proportion of CD4 + CD8 + DP cells in PBMCs from HAM-TSP patients vs. ACs and HCs	DP cells are strong IL-17 producers among T-cell lineage	(80)
		Higher mRNA levels of *IL17* in neutrophils from HAM-TSP patients vs. ACs	Along with increased Nf-kB-related genes	(85)
TNF (α/β)	TNFSF 1,2	Tax-treated MDDCs secrete TNF-a and -0 cytokines in culture	Cell supernatants (24/48 h of culture); induction in a Nf-KB-dependent manner	(36, 37)
		Tax-treated microglia cells secrete high protein levels for TNF-a	Cell supernatants (48 h of culture)	(39)
		Higher mRNA levels of *IL17* in PBMCs from ATLL patients vs. HCs	Along with increased Nf-KB-related genes	(51)
		Higher sera/plasma levels for TNF-a in HAM-TSP vs. ACs		(53)
		Higher plasma levels for TNF-a in ATLLpatients with aggressive cancer form vs. “indolent” form	Correlation between high plasma TNF-a levels and shorter survival rates in ATLL	(67)
		Increased proportion of CD4 + CD8 + DP cells in PBMCs from HAM-TSP patients vs. ACs and HCs	DP cells are strong TNF-a producers among T-cell lineage	(80)
		Higher TNF-a expression in CD14^+^monocytes from HAM-TSP vs. HCs	24 h of unstimulated PBMC culture	(82)
		Maintenance of high TNF-a production by CD14^+^CD16^+^ monocytes in HAM-TSP	Maintenance despite GM-CSF and IL-4 DC-driven differentiation	(83)
		Higher mRNA levels of *TNFA* in neutrophils from HAM-TSP patients vs. ACs	Along with increased Nf-kB-related genes	(85)
		HTLV-l-infected CD4T-cells from HTLV-1^+^ uveitis patients produced large amounts of TNF-a	Infiltrating CD4T-cells in eyes	(91)
		Increased TNF-a expression in FoxP3^+^ splenocytes from HBZ transgenic mice	Also higher IL-2 and IL-17 expressions in FoxP3 + cells from HBZ transgenic mice	(107)
IFN-γ	IFNG; IFG	Tax-treated MDDCs secrete IFN-y cytokine in culture	Cell supernatants (24/48 h of culture)	(36)
		Higher sera/plasma levels for IFN-y in HAM-TSP vs. ACs	Correlation between IFN-y and IL-6 levels in HAM-TSP patients	(53)
		Higher IFN-y production in PBMC supernatant of HAM-TSP patients vs. ACs (3 days if unstimulated culture)	Correlation between IFN-y and CXCL9/or CXCL10 levels in HAM-TSP patients	(66)
		Higher plasma levels for IFN-y in HAM-TSP patients vs. ACs	Higher levels in HAM-TSP patients vs. ATLL	(67)
		Higher IFN-y production in Tax-stimulated PBMCs in HAM-TSP patients vs. ACs and HCs	Higher IFN-y levels in ACs vs. HCs	(77)
		Higher plasma and mRNA levels (within PBMC) for IFN-y in HAM-TSP patients vs. Acs and HCs	Higher IFN-y levels in ACs vs. HCs (plasma and mRNA levels in PBMCs)	(78)
		Higher proportion of IFN-y-expressing CD8T-cells in HAM-TSP patients vs. ACs and HCs	PBMC culture with 3 days of cell stimulation	(79)
		Increased proportion of CD4 + CD8 + DP cells in PBMCs from HAM-TSP patients vs. ACs and HCs	DP cells are strong IFN-y producers among T-cell lineage	(80)
		Higher proportion of IFN-y-expressing CD56^h^′^8h^CD16′NKs in HAM-TSP patients vs. ACs	PBMCs stimulated for 48 h of culture with TLR_7/8_ agonist (innate sensing)	(84)
		HTLV-l-infected CD4T-cells from HTLV-1^+^ uveitis patients produced large amounts of IFN-y	Infiltrating CD4T-cells in eyes	(91)
		Increased IFN-y expression in both FoxP3^+^ and FoxP3’splenocytes from HBZ transgenic mice	Higher IFN-y-producing cells in both lung and PBMCs in HBZ transgenic mice	(107)

CTLA-8, cytotoxic T-lymphocyte-associated protein 8; IFN-y, interferon gamma; IL, interleukin; LAF, lymphocyte activatory factor; TNFSF, tumor necrosis factor superfamily.

**TABLE 2:** Pro-inflammatory chemokines and HTLV-1 infection, including associations with virus-related HAM-TSP and ATLL conditions.

Name	Aliases	Info in the context of HTLV-1 infection	Additional info	References
***Pro-inflammatory chemokines***				
MCP-1	CCL2	Tax-treated MDDCs secrete MCP-1 chemokinein culture	Cell supernatants (24/48 h of culture); also induction of CCLU (eotaxin)	(36)
		ATLL cells and HTLV-l-infected CD4 T-cell lines vs. uninfected cells display higher mRN A levels for *MCP1*	Tax- and Nf-KB-dependent process	(38)
		Higher sera and CSF protein levels for MCP-1 in both HAM-TSP patients and ACs vs. HCs	Also increased sera/CSF levels in HAM-TSP vs ACs for CCLU, CCL17 and CXCL5	(72)
		Increased mRNA expression in lung cells for *MCP1* in Tax transgenic mice vs. WT animals	Along with increased nRNA levels for pro-inflammatory cytokines *(IL1B, TNFA* and *IFNG)*	(94)
MIP-lα/β	CCL3 (MIP-lα) and CCL4 (MIP-1β)	Tax-treated MDDCs secrete both MIP-lα and -β chemokines in culture	Cell supernatants (24 h of culture); MIP-lα induction in a Nf-KB-dependent manner	(37)
		Tax-treated PBMCs induce both MIPl-α and -β secretions in culture su pern anta nt	Nf-KB-dependent processes; 2–24 h of culture	(40, 41)
		Increased CSF levels for both MIPlα/β in HAM-TSP vs. ACs	No difference in sera levels	(72)
		Higher proportion of MIP-lα-expressing monocytes and pDCs in HAM-TSP patients vs. ACs	PBMCs stimulated for 48 h of culture with TLR_7Z8_ agonist (innate sensing)	(84)
		High BALF levels for MIP-lα in HTLV-l-infected patients vs. HCs	Correlation between MIP-lα levelsand% of activated T-cells in BALFfrom HTLV-1* patients with CFA	(93)
		Increased mRNA expression in lung cells for *MIP1A* and *IB* inTax transgenic mice vs. WT animals	Along with increased nRN A levels for pro-inflammatory cytokines *(IL1B, TNFA* and *IFNG)*	(94)
		Increased supernatant secretion for MIP-lα in HTLV-1^+^CD4 T-cell lines vs. HTLV-1’ cells	Tax-dependent manner process	(103)
RANTES	CCL5	ATLL cells and HTLV-l-infected CD4T-cell lines vs uninfected cells display higher mRNA levels for *RANTES*	Tax- and Nf-KB-dependent process	(38)
		Tax-treated PBMCs induce RANTES secretion in culture supernantants	Nf-KB-dependent processes	(40, 41)
		Higher sera and CSF levels for RANTES in HAM-TSP patients vs. ACs		(53, 71)
		Higher RANTES release by immature MDMs from HAM-TSP patients vs. ACs and HCs	Culture supernantant (48 h of unstimulated culture)	(81)
		. Increased mRNA expression in lung cells for *RANTES* in Tax transgenic mice vs. WT animals	Along with increased nRNA levels for pro-inflammatory cytokines *(IL1B, TNFA* and *IFNG)*	(94)
		Increased supernatant secretion for RANTES in HTLV-1* CD4 T-cell lines vs. HTLV-1’cells	Tax-dependent manner process	(103)
		Increased secretion for RANTES a dn intracellular mRNA levels in HTLV-1* CD4T-cell lines vs. HTLV-1 cells	Tax- and Nf-KB-dependent manner process	(104)
MCP-3	CCL7	Tax-treated MDDCs secrete MCP-3chemokinein culture	Cell supernatants (24/48 h of culture); also induction of CCLU (eotaxin)	(36)
CXCL8	IL-8	Higher sera and CSF protein levels for IL-8 in both HAM-TSP patients and ACs vs HCs	Also increased sera/CSF levels in HAM-TSP vs. ACs for CCL11, CCL17 and CXCL5	(72)
		Increased supernatant secretion for IL-8 in HTLV-1* CD4 T-cell lines vs. HTLV-1’ cells	Tax-dependent manner process	(103)
CXCL9	MIG	Higher sera and CSF levels for CXCL9 in HAM-TSP patients vs. ACs and HCs	Similar levels between ACs and HCs; CXCL9 levels strongly correlates with disease progression	(53, 66, 71, 72)
		Higher CXCL9 release by immature MDMs from HAM-TSP patients vs. ACs and HCs	Culture supernantant (48 h of unstimulated culture)	(81)
CXCL10	IP-10	Higher sera and CSF levels for CXCL10 in HAM-TSP patients vs. ACs and HCs	Increased levels in ACsva HCs; CXCL10 levels strongly correlates with disease progression	(53, 66, 68, 71, 72)
		Higher plasma levels for CXCL10 in HAM-TSP patients vs. ACs and ATLL	Trend of higher CXCL10 levels with aggressive forms of ATLL vs. “indolent” form	(67)
		Higher CSF levels for CXCL10 in HAM-TSP patients vs. ACs	Higher CXCL10 levels in deteriorating patients with loss of motor function (weelchair)	(70)
		Increased proportion of CD4 + CD8 + DP cells in PBMCs from HAM-TSP patients vs. ACs and HCs	DP cells are strong CXCL10 producers among T-cell lineage	(80)
		High BALF levels for CXCL10 in HTLV-l-infected patients vs. HCs	Correlation between CXCL10 levels and% of activated T-cells in BALFfrom HTLV-1* patients with CFA	(93)
		Increased supernatant secretion for CXCL10 in HTLV-1* CD4T-cell lines vs. HTLV-1’ cells	Tax-dependent manner process	(103)
CXCL11	l-TAC	Higher sera and CSF levels for CXCLllin HAM-TSP patients vs. ACs and HCs	Similar levels between ACs and HCs	(53, 66, 71, 72)

CCL, C-C motif chemokine ligand; CXCL, C-X-C motif chemokine ligand; IP-10, interferon-induced protein 10; l-TAC, interferon-inducible T-cell alpha chemoattractant; MCP, monocyte chemoattractant protein; MIG, monokine induced by gamma interferon; MIP, macrophage inflammatory protein; RANTES, regulated on activation, normal T-cell expressed and secreted.

### 2.1 Impact of HTLV-1 tax protein

Like other retroviruses, the integrated HTLV-1 proviral genome is made up of two long terminal repeat sequences, flanking structural genes *gag*, *pol*, and *env*. HTLV-1 also has a unique 1.6 kb accessory region, termed the pX region, which encodes a few regulatory viral proteins when cells are productively infected (14, 20, 21). These mainly include the expression of the trans-activator protein Tax, which is known to hijack multiple intracellular signaling pathways that contribute to inflammation and immune activation, thereby ultimately promoting the proliferation of HTLV-1-infected T-cells and viral dissemination (22, 23). Among those, the nuclear transcription factor NF-κB plays a central role in coordinating various cellular signals that serve as pivotal mediators of inflammatory responses in the form of multiple encoding cytokines and chemokines (IL-1β, IL-2, IL-6, IL-8, TNF-α, MIP-1α/β and RANTES among others) (22, 24–26). The prototypical NF-κB complex corresponds to a heterodimer of the NF-κB1 (p50) and RelA (p65) members of the NF-κB/Rel family of transcription factors (27). Evidence shows that HTLV-1 Tax has developed multiple hijacking strategies to activate NF-κB signaling pathway; First, it induces the phosphorylation and degradation of both IκBα and IκBβ through the activation of the IκB kinase (IKK) complex, resulting in the nuclear translocation of active NF-κB (27–29). Tax also recruits the co-activator protein p300/CBP (30, 31) whose nuclear interaction with the RelA subunit of NF-κB is vital for RelA-dependent gene transcription (32). Finally, Tax stimulates the catalytic activity of the IKK-activating kinase TAK1 and mediates the physical recruitment of IKK to TAK1

in productively infected cells, including Tax-positive HTLV-1-transformed T-cells (33–35). Evidence shows that HTLV-1 Tax induces the secretion of multiple pro-inflammatory cytokines (IL-2, IL-12, TNF-α/β, and IFN- γ) and chemokines (MCP-1, MIP-1α/β, and MCP-3) in immature monocyte-derived dendritic cells (MDDCs) in a NF-κB-dependent manner (36, 37). Jurkat CD4 T-cell line, when treated with Tax, induces transactivation of the *MCP1* gene (38). Both peripheral monocyte-derived macrophages (MDMs) and microglia (specialized cells, acting as the brain’s resident macrophages), when cultivated *in vitro* with HTLV-1 Tax, secrete high amounts of pro-inflammatory IL-1β, and IL-6, and TNF-α cytokines (39). Similarly, HTLV-1 Tax mediates MIP-1α/β and RANTES expression in peripheral mononuclear cells (PBMCs) via the NF-κB signaling pathway (40, 41). Finally, although Tax expression in HTLV-1-infected individuals is tightly regulated and often silenced to evade immune detection, especially in ATLL patients, it can still be reactivated by multiple stressors, such as hypoxia, T-cell reactivation and oxidative stress, thereby sustaining viral persistence and long-lasting proinflammatory immune responses (42–44). So far, HTLV-1 Tax is one of the key viral proteins which has comprehensive executive function associated with developing HAM-TSP and ATLL conditions, that especially contribute in tissue inflammation/damage and T-cell hyperimmune activation (24, 45–48).

### 2.2 Impact of host innate sensing and autocrine regulation by TNF-α

Innate immune-mediated inflammation plays a critical role in inhibiting pathogenic viruses through the recognition of multiple viral components by the host pattern recognition receptors (PRRs) (49). In the context of HTLV-1 infection, the replication cycle yields multiple pathogen-associated molecular patterns such as viral RNA, RNA/DNA intermediates, and single- or double-stranded DNA, which are recognized by cytosolic sensors including cGAS, IFI-16, along with Ku70, which activate the STING-TBK-1 axis to induce IRF3-driven type I interferon responses. Endosomal PRRs such as toll-like receptor 3 (TLR3), TLR7/8, and TLR9 also respond to viral RNA or CpG-rich DNA via TRIF- or MyD88-dependent pathways, converging on both IRF3 and NF-κB inflammatory signaling (50). Surface TLRs such as TLR2 and TLR4 actively participate in sensing; For example, the accessory protein HTLV-1 p30 antagonizes TLR4 signaling in monocytes and dendritic cells, thereby deregulating MCP-1, TNF-α, IL-8 and IL-10 production (51). Its regulatory protein Tax robustly activates NF-κB and AP-1 transcription factors, driving expression of IL-2, IL-6, TNF-α, CCL2/MCP-1, and CXCL10 (47, 52), whereas HTLV-1 HBZ protein attenuates IRF3-mediated interferon signaling, dampening IFN-I responses (53). Additionally, HTLV-1 p12 and p8 proteins can both modulate IL-2 receptor signaling and enhance STAT5 activation even in the absence of IL-2, while facilitating immune evasion and cell-to-cell transmission (54, 55). Collectively, this complex interplay of innate sensing and viral countermeasures orchestrates a potent inflammatory and chemotactic cytokine milieu -comprising IL-1β, IL-2, IL-6, TNF-α, CCL2, CXCL10, and RANTES - that underlies chronic inflammation in HTLV-1–associated diseases such as ATLL and HAM-TSP (16, 50). Although there are intricate strategies employed by HTLV-1 to subvert the host innate IFN-I responses that contribute both to viral immune evasion and the development of HTLV-1-associated diseases (50), chronic HTLV-1 infection is still associated with unrelenting host innate sensing and immune activation accompanied by high levels of pro-inflammatory cytokines/chemokines (56–59). This outcome likely results from the host’s continuous, albeit unsuccessful, attempts to eradicate viral infection and maintain tissue homeostasis. In this context, the pro-inflammatory TNF-α cytokine, known for triggering upstream signaling events leading to the autocrine activation of NF-κB pathway (60), is likely one of the critical players of sustained HTLV-1-driven inflammation. Recent studies have shown that anti-TNF-α agents can be effective in treating inflammatory diseases related to HTLV-1 (such as arthropathy, uveitis, and other rheumatic conditions associated with the given viral infection) (61–64). In fact, the inflammatory response triggered by the increased of TNF-α along with IFN-γ and IL-2, mainly by the CD4 T-cell response, is what maintains the chronic inflammatory process in HAM-TSP patients (65–67).

## 3 Strong pro-inflammatory signatures in HAM-TSP and ATLL patients

Evidence shows that both aberrant expression and/or function of pro-inflammatory cytokines and chemokines actively contribute to the pathogenesis of HTLV-1-associated diseases involved in the inflammation of the central nervous system (CNS), which occurs in cases of HAM-TSP, as well as T-cell immortalization and tissue infiltration observed in ATLL patients (19, 68, 69) (Figure 1 and Tables 1, 2). In addition to immune activation, chronic HTLV-1–driven inflammation may also foster a pro-angiogenic microenvironment. Tax-mediated NF-κB activation stimulates the expression of vascular endothelial growth factor (VEGF) and basic fibroblast growth factor (bFGF), potent mediators of angiogenesis. These factors promote endothelial proliferation, vascular remodeling, and increased permeability changes that may facilitate tumor cell migration to lymph nodes in ATLL. Thus, angiogenesis represents a complementary mechanism through which HTLV-1–induced inflammation may contribute to disease progression (70, 71).

### 3.1 Profile in HAM-TSP

HTLV-1-associated myelopathy/tropical spastic paraparesis is a progressive disease of the CNS that causes weakness or paralysis of the legs, lower back pain, and urinary symptoms, which occur in approximately 2%–3% of HTLV-1 carriers (72). This HTLV-1-related disease is characterized by a hyper-stimulated immune response, which includes elevated levels of inflammatory cytokines and chemokines, and the recruitment/oligonal expansion of virus-specific cytotoxic CD8 T-cells in the CNS; all of which contributes to nerve tissue damage and loss of motor functions in patients. In this context, data show that the sera/plasma and cerebrospinal fluid (CSF) from HAM-TSP patients always display higher protein levels of IL-2, IL-6, IL-17, TNF-α, IFN-γ, MCP-1, RANTES, CXCL9, CXCL10, and CXCL11 when compared to ACs (58, 73–80). Data also show increased *ex vivo* levels of CSF neopterin in HAM-TSP patients (77, 79–81), which is a molecule synthesized by macrophages upon stimulation with IFN-γ and is indicative of a pro-inflammatory immune status (82). In fact, CSF CXCL9, CXCL10, and neopterin are described as trustworthy prognostic biomarkers for HAM-TSP disease progression (77–79, 81, 83). Although no differences are detected between HAM-TSP patients and ACs, levels of MCP-1 and IL-8 chemokines are higher in the sera and CSF from HTLV-1-infected individuals when compared to HCs (79). Research studies have further confirmed the higher cellular ability of HAM-TSP patients to secrete pro-inflammatory cytokines and chemokines in culture. For example, peripheral blood mononuclear cells (PBMCs) and CD8 T-cells of HAM-TSP patients stimulated or not with Tax peptides display higher mRNA and protein levels of IFN-γ when compared to both ACs and HCs (84–86). PBMCs from HAM-TSP patients further show an increased proportion of IL-17-expressing CD4 T-cells (86) and of inflammatory CD4^+^CD8^+^ T-cell populations whose IFN-γ, TNF-α, IL-17, and CXCL10 productions are one of the most pronounced among stimulated T-cells (87). Immature MDMs from HAM-TSP patients, when incubated in unstimulated cultures, show higher spontaneous secretion of RANTES and CXCL9 chemokines in comparison to both ACs and HCs (88). It is worth noting that pro-inflammatory CD16^+^ monocytes of HAM-TSP patients, whose proportions are increased in PBMCs in comparison to both ACs and HCs (88), are unable to fully mature into dendritic cells and maintain a high production of TNF-α and IL-1β cytokines (89, 90). Similarly, higher proportions of pro-inflammatory monocytes producing IL-12 and MIP-1α, plasmacytoid DCs producing IL-12 and CD56*^high^*CD16^–^ natural killer cells producing IFN-γ are detected in HAM-TSP blood samples in response to innate immune sensing when compared to ACs (91). The comparative gene expression profiles of polynuclear neutrophils between ACs and HAM-TSP patients have revealed higher expression of multiple genes related to the Nf-kB signaling pathway and pro-inflammatory responses including *TNFA*, *IL6*, and *IL17* (92). Altogether, this evidence infers that, during HAM-TSP development model, HTLV-1-infected cells in the CNS may produce large amounts of IFN-γ that can induce resident macrophages, DCs, neutrophils and astrocytes to secrete MCP-1, MIP-1α/β, RANTES, CXCL9, and CXCL10 chemokines among others (7, 93–95). The latter recruit more infected cells, including Tax-expressing CD4 T-cells, to the aera along with cytotoxic CD8 T-cells, which constitute a T-helper type 1 (Th1)-centric feedback loop (IL-1β, IL-2, TNF-α, IL-12 and IFN-γ) that results in chronic inflammation in the CNS (95–97).

### 3.2 Profile in other HTLV-1-associated inflammatory conditions

In addition to the HAM-TSP disease, HTLV-1 infection can cause inflammation in other tissues than CNS. In this context, HTLV-1-infected CD4 T-cells collected from patients suffering from uveitis, which is the second-most frequent HTLV-1-associated disease in Japan after HAM-TSP, also produce large amounts of various inflammatory cytokines such as IL-1, IL-6, TNF-α, and IFN-γ (98, 99). Similarly, evidence shows that the bronchoalveolar fluids (BALFs) from HTLV-1-infected patients with CFA, a chronic inflammatory lung disease of unknown etiology, display elevated levels of MIP-1α and CXCL10 chemokines, which correlate with higher tissue infiltration of activated T-cells (100, 101). Altogether, this indicates that, similarly to HAM-TSP, HTLV-1 infection may contribute to other HTLV-1-associated inflammatory diseases via the chemokine-dependent recruitment of activated T-cells in the eyes (uveitis) and lungs (CFA), thus resulting in chronic tissue inflammation by the sustained release of Th1 pro-inflammatory cytokines.

### 3.3 Profile in ATLL

Adult T-cell leukemia/lymphoma is a highly aggressive mature CD4^+^CD25^+^FoxP3^+^ T-cell neoplasm associated with chronic HTLV-1 infection, which affects around 10 million people worldwide. Although it is obvious that HTLV-1-associated inflammatory conditions (HAM-TSP, uveitis, and CFA) are associated with elevated pro-inflammatory innate and T-cell immune responses, ATLL patients exhibit a rather immunosuppressive profile that is mainly highlighted by the abnormally high production of IL-10 cytokine (102). However, ATLL is a complex and multistep disease, which involves HTLV-1 Tax and HBZ proteins and starts with high proliferation and survival of HTLV-1-infected CD4 T-cells (21, 103). In fact, one of the major hallmarks of HTLV-1-infected CD4 T-cells in ATLL is their ability to proliferate independently of T-cell receptor stimulation, contributing to the immortalization of these infected cells overtime (6, 21, 69). Although survival of HTLV-1-infected ATLL CD4 T-cells mainly depend on IL-10 production (74, 102, 104), evidence shows that the maintenance of elevated proliferation rates is rather supported by their dependency on pro-inflammatory cytokines such as IL-2 and IL-6. In this context, *in vitro* data confirm that HTLV-1-infected T-cells are dependent on IL-2 for their proliferation, until they get their immortalized status after several weeks in culture (105). Levels of IL-6 in sera are higher in ATLL patients when compared to both ACs and HCs (106, 107). Interestingly, IL-6 levels in ATLL do not only correlate with T-cell proliferation but also with ATLL severity and shorter survival rate in patients (107). Recent comparative transcriptomic analyses of PBMCs between ATLL patients and HCs have revealed that ATLL pathogenesis is associated with the upregulation of many genes related to inflammatory responses such as *NFKB1*, *RELA*, *IL2, IL17*, and *TNFA* (56, 108, 109). Pro-inflammatory chemokines are also involved in ATLL pathogenesis as they recruit cancer cells into the lymph nodes, spleen, liver, skin and gastrointestinal tract, thereby contributing to cancer spreading in infected patients (63, 69). In fact, constitutive expression of various pro-inflammatory chemokines in HTLV-1-positive ATLL cells, including MCP-1, MIP-1α/β, RANTES, IL-8 and CXCL10, have been reported and involves the HTLV-1 Tax and Nf-κB signaling pathway in the process (38, 110, 111). Higher plasma levels of TNF-α and IL-6 cytokines, and CXCL10 are found in patients with aggressive ATLL when compared to those with indolent ATLL (*aka.* stable and slow growing form of lymphoma/leukemia usually associated with lesser fever and lesser symptoms), indicating a worsening role of strong inflammatory responses in ATLL disease severity (74, 107). Finally, Tax- and HBZ-transgenic mice, which develop HTLV-1-like impairments such as T-cell lymphoma and systemic inflammation, display higher production of TNF-α and IFN-γ in FoxP3^+^ splenocytes, and of IL-1α/β and IL-6 in ATLL-like cells (112–114).

## 4 Conclusive remarks

Overall, it is obvious that the clinical burden and lack of effective treatment options directs the need for alternative treatment strategies for HTLV-1 infection (14). In this context, a more refined understanding of how HTLV-1 infection, in the presence or absence of Tax protein, influences the sustained pro-inflammatory cytokine/chemokine host production is key for identifying new mechanisms underlying HTLV-1 persistence and development of more effective therapies against HTLV-1-associated diseases (Figure 1).
